# Hardware Acceleration of Division-Free Quadrature-Based Square Rooting Approach for Near-Lossless Compression of Hyperspectral Images

**DOI:** 10.3390/s25041092

**Published:** 2025-02-12

**Authors:** Amal Altamimi, Belgacem Ben Youssef

**Affiliations:** 1 Space Technologies Institute, King Abdulaziz City for Science and Technology, P.O. Box 8612, Riyadh 12354, Saudi Arabia; aaltamimi@kacst.gov.sa; 2 Department of Computer Engineering, College of Computer and Information Sciences, King Saud University, P.O. Box 51178, Riyadh 11543, Saudi Arabia

**Keywords:** hyperspectral imaging, data compression, hardware acceleration, FPGA, near-lossless compression, real-time processing, quadrature-based square rooting, power, efficiency, throughput

## Abstract

Recent advancements in hyperspectral imaging have significantly increased the acquired data volume, creating a need for more efficient compression methods for handling the growing storage and transmission demands. These challenges are particularly critical for onboard satellite systems, where power and computational resources are limited, and real-time processing is essential. In this article, we present a novel FPGA-based hardware acceleration of a near-lossless compression technique for hyperspectral images by leveraging a division-free quadrature-based square rooting method. In this regard, the two division operations inherent in the original approach were replaced with pre-computed reciprocals, multiplications, and a geometric series expansion. Optimized for real-time applications, the synthesis results show that our approach achieves a high throughput of 1611.77 Mega Samples per second (MSps) and a low power requirement of 0.886 Watts on the economical Cyclone V FPGA. This results in an efficiency of 1819.15 MSps/Watt, which, to the best of our knowledge, surpasses recent state-of-the-art hardware implementations in the context of near-lossless compression of hyperspectral images.

## 1. Introduction

Notable developments in digital imaging technologies have significantly improved the capabilities of hyperspectral imaging (HSI). These systems are capable of capturing images across an extensive range of wavelengths and producing substantial datasets that are enriched with high-resolution spectral information for each pixel. The depth and precision of these data prove essential in diverse fields, such as remote sensing [1,2], medical diagnostics [3,4,5], agricultural monitoring [6,7], geological exploration [8,9], water resource management [10,11], and urban planning [12], thus providing critical insights that are unattainable with conventional imaging techniques. With the massive data produced by hyperspectral imaging systems, efficient data compression becomes critical. The challenge lies in compressing these data effectively to reduce storage demands and facilitate faster data transmission without losing the integrity and quality of the spectral information. This necessity drives the development of innovative compression methods that can handle the complexity and size of hyperspectral data.

Traditional compression algorithms have primarily been software-based and utilize standard techniques that often struggle with the high data rates and volume generated by modern hyperspectral sensors [13,14,15]. These software solutions typically operate on general-purpose processors, which can limit their efficiency and speed due to the computational overheads and the need for multiple processing cycles [16]. As software-based compression techniques often fall short in meeting the real-time processing requirements of hyperspectral imaging, there is a growing interest in hardware-accelerated solutions [17]. Field-Programmable Gate Arrays (FPGAs) and other dedicated hardware platforms offer the potential to accelerate compression tasks, providing the necessary speed and flexibility. Hardware acceleration can significantly enhance processing capabilities, enabling real-time data compression and analysis [18,19,20,21].

In satellite applications, the efficiency of data compression is particularly crucial. These platforms face unique challenges such as the limited availability of power and the need to manage vast amounts of data efficiently within constrained onboard processing capabilities. Effective compression algorithms must, therefore, minimize power requirement and maximize throughput to ensure timely and efficient data handling and transmission from orbit [22].

High throughput and compression ratios are often achieved using lossy compression techniques, which reduce data size significantly but at the cost of introducing artifacts and potentially losing important details. On the other hand, lossless compression methods preserve data integrity, ensuring no loss of information despite suffering from low throughput and less efficient compression ratios. Near-lossless compression offers a balanced trade-off by striving to achieve reasonable performance across all these metrics. It significantly reduces data size while maintaining high fidelity and minimal loss of critical information, thus limiting the pixel distortion to a pre-defined absolute or relative error [23,24,25,26,27]. A stricter definition limits the maximum error to the intrinsic noise of the original data produced by the instrument or other sources, which is similar to atmospheric correction [28]. Near-lossless compression is generally achieved by employing one of the following three approaches: lossless encoding of the prediction error after quantization; quantizing the original image first and then performing lossless encoding; or implementing a two-stage near-lossless encoding. The first approach is the most widely used technique due to its low complexity. Methods under this category include variations of the Context-Based, Adaptive, Lossless Image Coder (CALIC) [29,30,31] and others, which are based on the Consultative Committee for Space Data Systems (CCSDS) standards [32,33]. The second approach typically yields poor compression performance with increasing tolerance values [25]. The third approach employs both lossy and lossless compression techniques such as those proposed in [25,34]. Building on these advancements, this article makes the following key contributions:

- Introducing a division-free quadrature-based method that aims to achieve near-lossless HSI compression by reformulating the original algorithm to avoid two division operations. This method is optimized for speed and efficiency while ensuring high accuracy.
- Describing the hardware acceleration of HSI near-lossless compression while utilizing innovative seed generation and quadrature-based square rooting techniques.
- Achieving high-performance metrics targeting the Cyclone V FPGA. Synthesis results achieve a high throughput of 1611.77 Mega Samples per second (MSps) with a low power requirement of 0.886 Watts, yielding a notable efficiency value compared to existing state-of-the-art techniques.

The remainder of this paper is structured as follows: Section 2 reviews the existing literature on near-lossless hyperspectral image compression. Section 3 presents our near-lossless compression approach, including specifics about the seed generation technique and the quadrature-based square rooting method. Section 4 extends this approach by introducing a division-free approach detailing the elimination of two division operations from the original method. Section 5 focuses on the hardware implementation details of the proposed algorithm. Section 6 describes the performance evaluation of the division-free algorithm for near-lossless HSI compression by providing a detailed analysis of resource utilization, clock frequency, throughput, power requirement, and comparisons with state-of-the-art implementations. Finally, Section 7 concludes the paper with a summary of our key findings while suggesting directions for future research.

## 2. Related Work

Since our last systematic review [17], recent studies on hardware-accelerated near-lossless compression of hyperspectral images have demonstrated a focused interest in the Consultative Committee for Space Data Systems (CCSDS) standard, known as the CCSDS 123, particularly when employing FPGA technology. This alignment of studies demonstrates a collective endeavor to refine and enhance proven technologies to meet the stringent requirements of space-based imaging. The following summaries illustrate the contributions of these recent works in enhancing both the efficiency and performance of near-lossless compression.

In their 2021 study, Barrios et al. describe a hardware implementation of the CCSDS 123.0-B-2 near-lossless compression standard using a Xilinx Kintex UltraScale FPGA and a High-Level Synthesis (HLS) design approach [35]. The implementation is designed to manage the high data volumes from advanced imaging sensors on satellites, which are limited by onboard computational and storage capacities and narrow downlink bandwidths. The implemented system delivers a throughput of 12.5 MSps, utilizing 7% of the FPGA’s Look-Up Tables (LUTs) and approximately 14% of its dedicated memory blocks. The total power requirement of the system is about 2.48 Watts. Expanding on these findings, a subsequent study by Sánchez et al. explores enhancements to the CCSDS 123.0-B-2 standard by notably increasing throughput to 125 MSps without degrading compression performance [36]. The study addresses the challenge of significant data dependencies introduced by the feedback loop, particularly those due to the quantization stage, which hinders real-time processing. By analytically removing the quantization from the feedback loop, the authors effectively reduce the critical path, thus enhancing the overall operational throughput.

A recent study introduces an advanced design and implementation of near-lossless hyperspectral image compression tailored for high-throughput space missions [37]. Utilizing the CCSDS-123.0-B-2 standard, the described architecture addresses a performance bottleneck associated with internal closed-loop quantizers by adopting an external quantization method while maintaining near-lossless compression quality. Achieving a significant throughput of up to 1375 MSps through the utilization of a parallel processing approach, the system is implemented on a Xilinx Kintex Ultrascale XCKU040 FPGA. The power requirement for the entire compression engine is approximately 4.221 Watts.

In their work, Chatziantoniou et al. present an architecture that effectively implements the CCSDS-123.0-B-2 standard on a Xilinx Kintex Ultrascale XCKU040 SRAM FPGA [38]. The architecture utilizes a hybrid entropy coder, which significantly enhances compression performance at low bit rates compared to earlier entropy coders. Operating at one sample per cycle and achieving a constant throughput of 305 MSps, the system is designed to switch seamlessly between lossless and near-lossless modes. This implementation also maintains a low power requirement of 1.525 Watts.

A real-time FPGA implementation of the CCSDS 123.0-B-2 standard for hyperspectral image compression, using a Virtex-7 VC709 board with an XCKU040 FPGA, is detailed in [39]. This implementation features a deeply pipelined architecture with a frame interleaved by a diagonal (FID) traversal method that effectively manages data dependencies, achieving a processing efficiency of 0.9984 samples per cycle at a clock rate of 250 MHz. This translates to a processing throughput of approximately 249.6 MSps, where a low power requirement of 1.2 Watts is maintained. These studies collectively advance the state-of-the-art of FPGA-based hyperspectral image compression by demonstrating practical implementations of the CCSDS 123.0-B-2 standard on various FPGA platforms.

## 3. Near-Lossless Compression of HSI

In our previous work [40], we introduced lossless and near-lossless compression algorithms for remotely sensed hyperspectral images. In these algorithms, compression is realized by utilizing a mathematical property that the integer part of the square root of a number *x* typically requires about half as many bits as *x* itself [41]. In addition, this reduction in bit requirements results in lower entropy, leading to decreased randomness and increased predictability within the dataset. The lossless compression employs seed generation coupled with entropy encoding, achieving a significant reduction of up to 62% while maintaining low computational complexity when benchmarked against the Corpus dataset [42]. However, the inherent complexity of some hyperspectral images posed significant challenges to lossless compression. These particularly complex images exhibit a collective reduction percentage of 25.36%, as represented by their geometric mean. In response, our near-lossless method was primarily designed to augment the lossless compressor, improving the reduction percentage to approximately 39% for those challenging hyperspectral images.

In terms of computational efficiency, the lossless compressor demonstrates high performance in the hardware-accelerated lossless compression of hyperspectral images. It achieves significant gains in throughput and power efficiency due to its reduced computational demands [43]. On the other hand, the near-lossless compression method contains two division operations that introduce additional computational complexity, potentially slowing down the pipeline compared to the fully lossless approach. Building on that foundation, this article extends the previous work by presenting a division-free version of the algorithm optimized for hardware implementation. This modification aims to improve computational efficiency by avoiding costly division operations, simplifying hardware complexity, and reducing power requirement.

### Quadrature-Based Method

**Figure 1:**
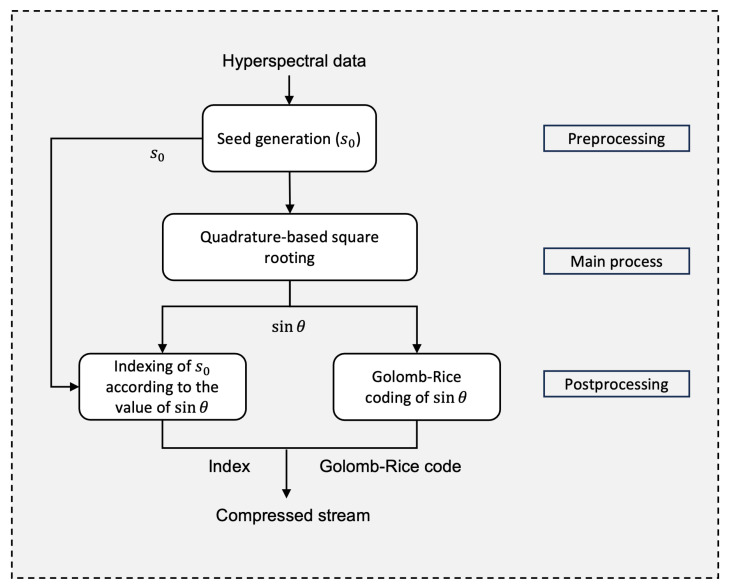
Flow diagram of the near-lossless compression of HSI employing the quadrature-based square rooting method.

The quadrature-based encoder used to achieve near-lossless compression is exhibited in Figure 1. Starting with hyperspectral data as input, the preprocessing step involves generating an initial estimate, $s_{0}$, of the square root for each 16-bit pixel value *x*. This estimate is obtained by averaging the leftmost ⌈n/2⌉ bits of *x*, i.e., the most significant half (MSH), with the scaled base $2^{\left\lfloor n/2\right\rfloor }$, where *n* is equal to the number of bits in *x*. That is, the seed $s_{0}$ can be computed by using the following formula:

(1) $$s_{0}=0.5\times \left(\mathrm{MSH}+2^{\left\lfloor n/2\right\rfloor }\right).$$

Unlike lossless compression, which employs bitwise XORing for decorrelation, this work on near-lossless compression does not employ spatial or spectral decorrelation as a preprocessing step. The quadrature-based square rooting method used here inherently produces some errors, which would lead to an error propagation that affects the quality of reconstructed data across those bands, when coupled with bitwise XORing for decorrelation. The potential for error amplification necessitates omitting bitwise XORing in the near-lossless approach, thereby making the method more universally applicable without relying on specific decorrelation strategies tailored to hyperspectral data. This is in contrast to the lossless compressor, where XORing between adjacent bands is completely reversible, allowing for exact data reconstruction.

**Figure 2:**
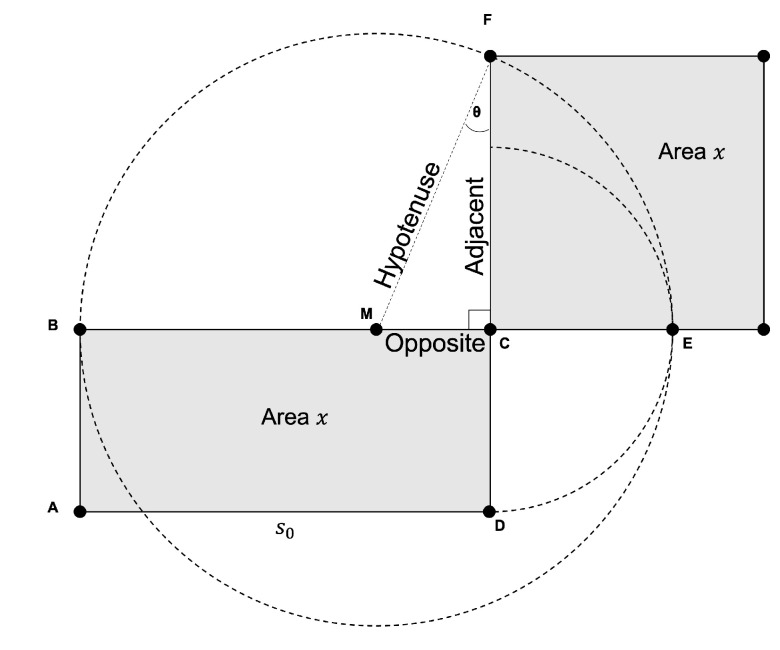
Geometric construction of a square with the same area *x* using the quadrature of rectangle ABCD.

For the main process, the quadrature-based square rooting method is employed to enhance the accuracy of the initial estimate of the square root value. Figure 2 illustrates the quadrature method by geometrically constructing a square of the same area *x* as the given rectangle ABCD, where $s_{0}$ represents one side of the rectangle.

The circle in the figure helps establish trigonometric relationships between the sides of the right triangle MCF, aiding in the computation of the square root. The adjacent side, shown in the figure and given by segment CF, corresponds to the desired square root value of *x*. The steps involved in the quadrature-based method for calculating the square root value are provided in Algorithm 1.
**Algorithm 1:** The quadrature-based square rooting method. 
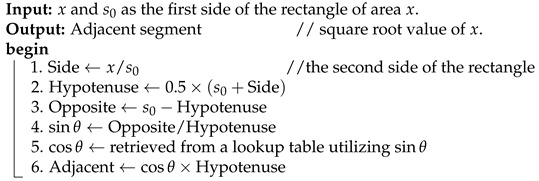


The accuracy of the initial estimate $s_{0}$ directly influences the angle θ between the hypotenuse and the adjacent side of the right triangle. When $\theta =0^{\circ }$, this translates to $s_{0}$ being an accurate estimate, and the quadrilateral we started with is effectively a perfect square. As the accuracy decreases, θ deviates from $0^{\circ }$, with the worst-case scenario occurring when θ approaches $\pm 90^{\circ }$. In these regards, simulation results in [44] demonstrate that $s_{0}$ constrains this deviation of θ to the range $\left[-30^{\circ },4^{\circ }\right)$. With a step size of 0.01, this corresponds to 57 possible values of sinθ within the closed interval [−0.5,0.06]. However, for pixel values of *x* up to $2^{16}-1$, only 29 of these values are actually accessed when $s_{0}$ is used [44].

The lengths of the hypotenuse and the adjacent segment are often represented with decimal precision, whereas $s_{0}$ is restricted to an integer value. To maintain an efficient compression system, we use $s_{0}$ alongside the quantized value of sinθ for encoding. Together, these two values provide sufficient information for the decoder to approximate *x*. This is achieved by squaring the product of the corresponding cosθ and the hypotenuse, where the latter is generated at the decoder by dividing $s_{0}$ by (1+sinθ). This equivalent value of the hypotenuse is derived next, using the notations presented in Figure 2:

$$\begin{array}{cc} & \sin\theta =\frac{\mathrm{Opposite}}{\mathrm{Hypotenuse}}\;\mathrm{and}\;\mathrm{Opposite}=s_{0}-\mathrm{Hypotenuse}\\ & \Rightarrow \sin\theta =\frac{s_{0}-\mathrm{Hypotenuse}}{\mathrm{Hypotenuse}}=\frac{s_{0}}{\mathrm{Hypotenuse}}-1\\ & \Rightarrow \mathrm{Hypotenuse}=\frac{s_{0}}{1+\sin\theta }. \end{array}$$

**Figure 3:**
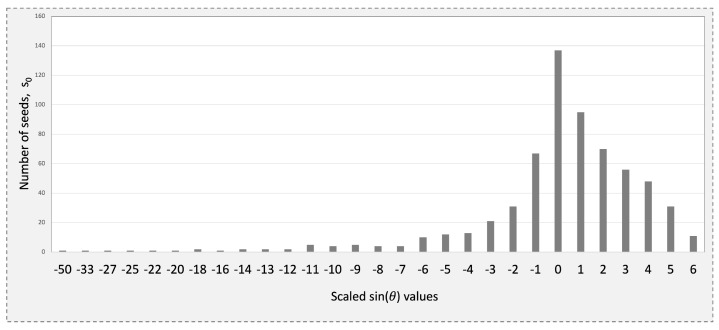
Skewed distribution of $s_{0}$ across the range of sinθ values for $x\in [0,2^{16}-1]$.

The postprocessing step aims to achieve a more compact representation by directly mapping shorter Golomb–Rice codes to sinθ values that correspond to larger sets of $s_{0}$ values [45]. This approach leverages the uneven distribution of $s_{0}$ across the range of sinθ values, as shown in Figure 3. Instead of storing the full representation of $s_{0}$, indexing is used to minimize storage requirements, ensuring that the number of bits does not exceed $1+\lfloor \log_{2}k\rfloor$, where *k* is the count of $s_{0}$ values for each sinθ. Ultimately, Golomb–Rice codes and the obtained indices are concatenated to produce the compressed stream.

## 4. Division-Free Quadrature

The steps detailed in Algorithm 1 involve two key division operations. The first division, given by the first pseudo-instruction, determines the length of the second segment (CD) of the rectangle with area *x*, assuming the length of the first segment (AD) is given by the previously generated seed $s_{0}$. The second division, included in the fourth pseudo-instruction, calculates the sine value by computing the ratio of the opposite segment to the hypotenuse of the right triangle. The following subsections describe the techniques used to bypass these two divisions.

### 4.1. Addressing the First Division

Given that the 8-bit seed value has a limited range from 0 to 255, we can optimize the quadrature process by precomputing the reciprocal of each seed value. These reciprocals can then be multiplied by the area *x* of the rectangle to efficiently compute the length of the second segment (CD). To ensure rapid access, these precomputed reciprocals are stored in an LUT that is directly addressed by the generated seeds.

### 4.2. Addressing the Second Division

The second division is formulated as sinθ=Opposite/Hypotenuse. In this context, the hypotenuse is the average of the two sides of the rectangle, while the opposite side represents the difference between this average and the generated seed. To avoid the division operation when calculating the value of sinθ, we use algebraic manipulation based on the following three relations:

$$\begin{array}{cc} \mathrm{Hypotenuse} & =0.5\times (s_{0}+x/s_{0}),\\ \mathrm{Opposite} & =s_{0}-\mathrm{Hypotenuse},\;\mathrm{and}\\ \sin\theta & =\mathrm{Opposite}/\mathrm{Hypotenuse}. \end{array}$$

Here, $s_{0}$ represents the first segment (AD) and $\left(x/s_{0}\right)$ corresponds to the second segment (CD). The total length of these two segments yields the diameter of the circle centered at point M. Thus, the Hypotenuse, which is equal to the corresponding radius, is obtained by averaging these two segments. Let *d* be the difference between the stated segments such that

$$d=s_{0}-\frac{x}{s_{0}},$$

which implies that

$$\frac{x}{s_{0}}=s_{0}-d.$$

Thus, we can redefine the Hypotenuse as follows:

$$\begin{array}{cc} \mathrm{Hypotenuse} & =0.5\times (s_{0}+s_{0}-d)\\ & =0.5\times (2s_{0}-d)\\ & =s_{0}-0.5d\;. \end{array}$$

The above leads to the following:

$$\begin{array}{cc} \sin\theta & =(s_{0}-\mathrm{Hypotenuse})/\mathrm{Hypotenuse}\\ & =\left(s_{0}-s_{0}+0.5d\right)/\left(s_{0}-0.5d\right)\\ & =0.5d/(s_{0}-0.5d)\;. \end{array}$$

Applying the principle that if two quantities are equal, their reciprocals are also equal, we obtain the following:

$$\begin{array}{cc} 1/\sin\theta & =(s_{0}-0.5d)/0.5d\\ & =s_{0}/0.5d-1\\ & =2s_{0}/d-1. \end{array}$$

By further manipulation of the above equation, we obtain the following:

$$\begin{array}{cc} 1+1/\sin\theta & =2s_{0}/d\;,\\ \left(\sin\theta /\sin\theta )+(1/\sin\theta \right) & =2s_{0}/d\;,\\ (1+\sin\theta )/\sin\theta & =2s_{0}/d\;, \end{array}$$

Since $1/s_{0}$ represents the precomputed reciprocal and is readily available, we can rewrite the above equation in a way that allows us to avoid the second division operation:

(2) $$\sin\theta /\left(1+\sin\theta \right)=2^{-1}\times d\times \left(1/s_{0}\right)\;.$$

Therefore, the division operation has been avoided in deriving the expression on the right-hand side of Equation (2). To recover the value of sinθ, let *Q* represent the right-hand side of the above equation:

sinθ/(1+sinθ)=Q

By inverting both sides, we obtain the following:

(1+sinθ)/sinθ=1/Q

We further expand and simplify this expression to obtain the following:

$$\begin{array}{cc} \left(1/\sin\theta )+(\sin\theta /\sin\theta \right) & =1/Q\\ (1/\sin\theta )+1 & =1/Q\\ \left(1/\sin\theta \right) & =(1/Q)-1\\ \left(1/\sin\theta \right) & =(1-Q)/Q\\ \sin\theta & =Q/(1-Q) \end{array}$$

The last equality approximates sinθ while maintaining division-free calculations by employing an iterative approach based on a truncated geometric series [46]. This method involves successive multiplications and additions to approximate the quantity Q/(1−Q) by summing a limited number of terms in the series $Q+Q^{2}+Q^{3}+\cdots +Q^{k}$ until the desired precision is achieved. Simulation results using MATLAB demonstrate that sinθ reaches the target accuracy after five terms in this series expansion. By the term target accuracy, we refer to the precision required to differentiate all 29 distinct values of sinθ.

At this stage, we have determined the required values of $s_{0}$ and sinθ, which are essential for the decoder to reconstruct the pixel value *x* without using any division operation.

### 4.3. Performance of Quadrature-Based Method

**Figure 4:**
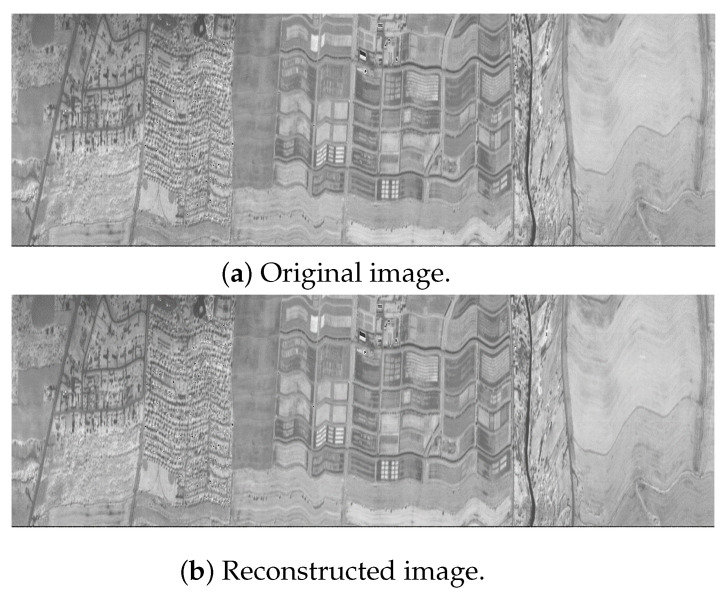
Near-lossless compression of CASI (t0477f06, U) band 70.

**Figure 5:**
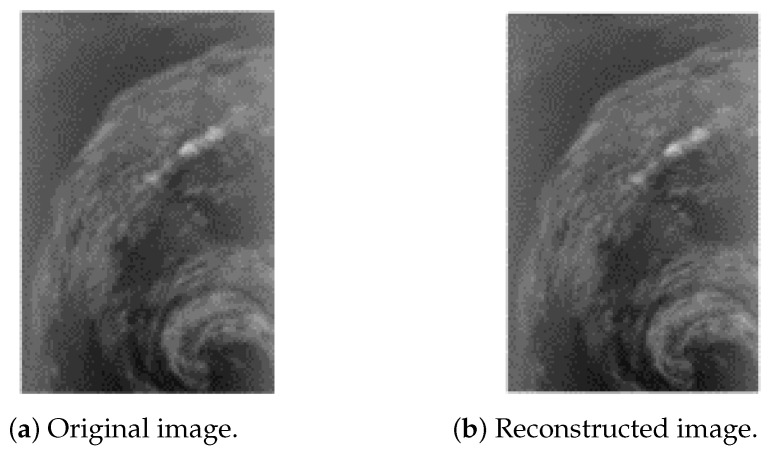
Near-lossless compression of AIRS (granule 16, U) band 208.

**Figure 6:**
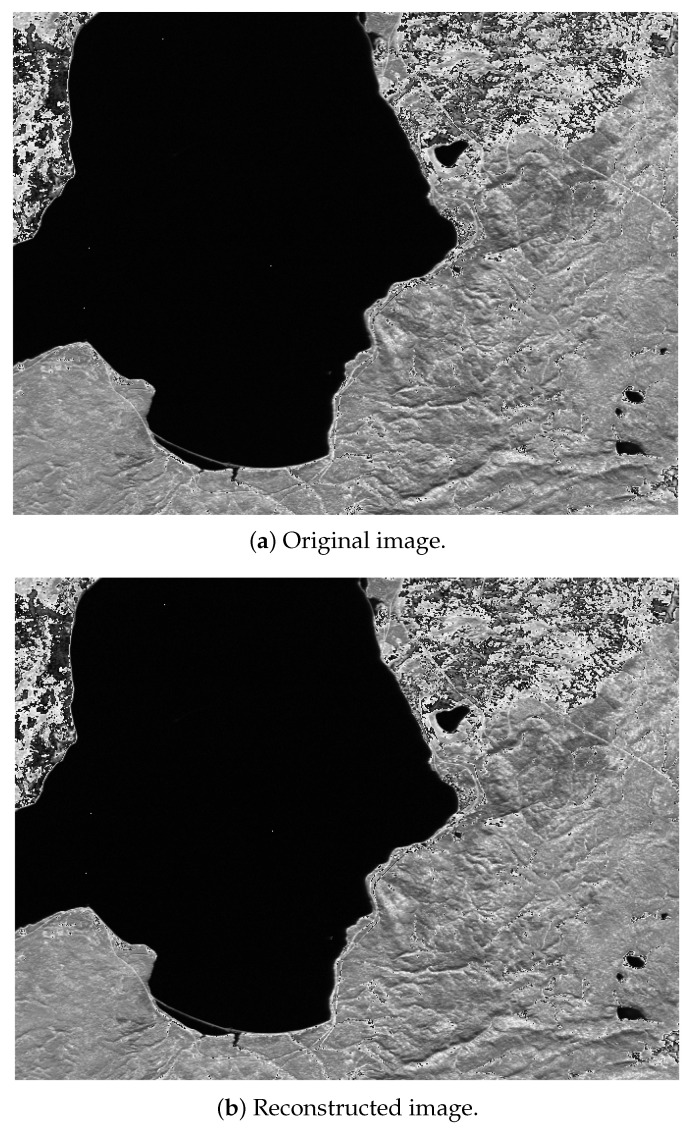
Near-lossless compression of AVIRIS Yellowstone (sc10, C) band 106.

Incorporating the quadrature-based square rooting method after seed generation significantly enhances the compression reduction rate in a near-lossless approach while maintaining high fidelity for the decompressed stream. Figure 4, Figure 5 and Figure 6 present the original and reconstructed images for the CASI uncalibrated image t0477f06 (band 70), the AIRS uncalibrated granule 16 image (band 208), and the AVIRIS calibrated Yellowstone image (band 106), respectively, after applying our near-lossless compressor. Visual assessment of these results indicates that each decompressed image closely resembles its original counterpart, demonstrating the high similarity achieved by the compression algorithm. The computed peak signal-to-noise ratio (PSNR) values for these three images are 50.2793 decibels (dB), 50.5717 dB, and 52.7601 dB, respectively. The maximum relative error across all images is 0.01, and the achieved compression reduction is 39% when rounded to the nearest integer. These hyperspectral images are taken from the publicly available Corpus dataset.

The performance of the near-lossless compressor on the aforementioned challenging images from the Corpus dataset is detailed in Table 1. The table highlights the improvement in reduction percentage compared to the results reported in [40] of the lossless method, along with the maximum relative error (MRE) and PSNR values achieved by the near-lossless approach.

## 5. Hardware Implementation

We describe herein the FPGA-based hardware structure optimized for quadrature-based, near-lossless compression of hyperspectral images. The design is divided into stages, each focusing on a specific part of the compression algorithm, as shown in Figure 1.

### 5.1. Preprocessing

The first stage of the compression system prepares the input data for the main process by calculating essential parameters and generating an initial estimate for the square root used as a seed value. This stage comprises two main components: the shift amount calculator, which determines the number of bit-shifts needed for the initial estimation of the square root, and the seed generation logic, which uses the resulting shift amount to produce this initial estimate.

#### 5.1.1. Shift-Amount Calculator

To apply our seed generation technique for approximating the square root value using Equation (1), we first need a dedicated logic to calculate the shift amount ⌊n/2⌋. This can be achieved through bit manipulation techniques such as counting the number of leading zeros in the unsigned binary representation of *x* to initially determine *n*. Many hardware platforms support these operations, facilitating faster computation of the bit length, *n* [47]. Typically, these methods have a computational complexity of O(n). Alternatively, employing a binary search to determine the binary logarithm of *x* results in a worst-case complexity of $O(\log_{2}x)$ [48]. Then, the count of bits required to represent a positive integer *x* in binary is equal to $1+\lfloor \log_{2}x\rfloor$ [49].

The binary search used to determine the shift amount requires four comparisons, which correspond to $\log_{2}16$, as the input data are processed in 16-bit chunks. Table 2 illustrates the pivots chosen for the binary search, which represent the maximum values for each bit length up to 16 bits. These pivots serve as the point of comparison to divide the search into smaller segments.

Algorithm 2 illustrates the use of the pivots listed in the table above within a binary search structure. The nested if–else conditions ensure that evaluating any given pixel value *x* requires no more than four comparisons.
**Algorithm 2:** Binary search algorithm for determining the shift amount for a 16-bit number *x*. 
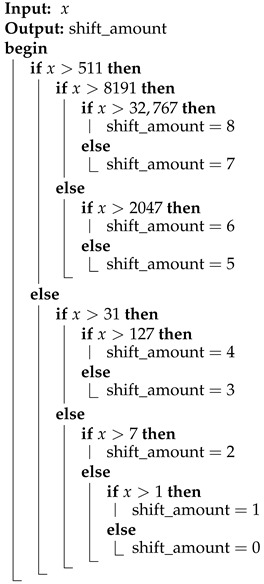


#### 5.1.2. Seed Generation Logic

**Figure 7:**
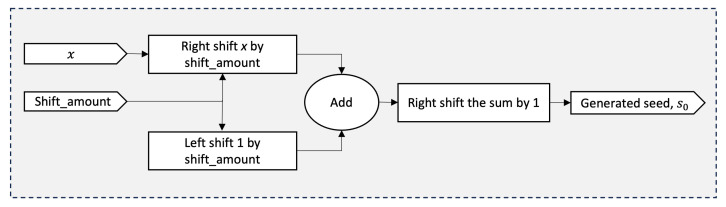
Block diagram showing the computation process of the initial estimate of the square root value, $s_{0}$.

Next, the seed generation block processes a 16-bit input *x* to produce the corresponding seed value, as dictated by the precalculated shift amount. On each rising clock edge, the block first performs a right-shift operation on *x*, extracting its most significant half. Concurrently, it generates the scaled base $2^{\left\lfloor n/2\right\rfloor }$ by left-shifting the constant 1 by the same shift amount. To clarify, raising the constant 2 to a power, say *k*, is equivalent to multiplying 1 by 2, *k* times. In other words, it corresponds to left-shifting the number 1 by *k* positions in binary representation. Once the MSH and the scaled base are computed, their sum is right-shifted to derive the final seed value. Given that the input value is limited to 16 bits, the resulting seed value is constrained to 8 bits. Figure 7 illustrates the graphical representation for calculating the estimated square root value for a 16-bit number.

### 5.2. Main Process

**Figure 8:**
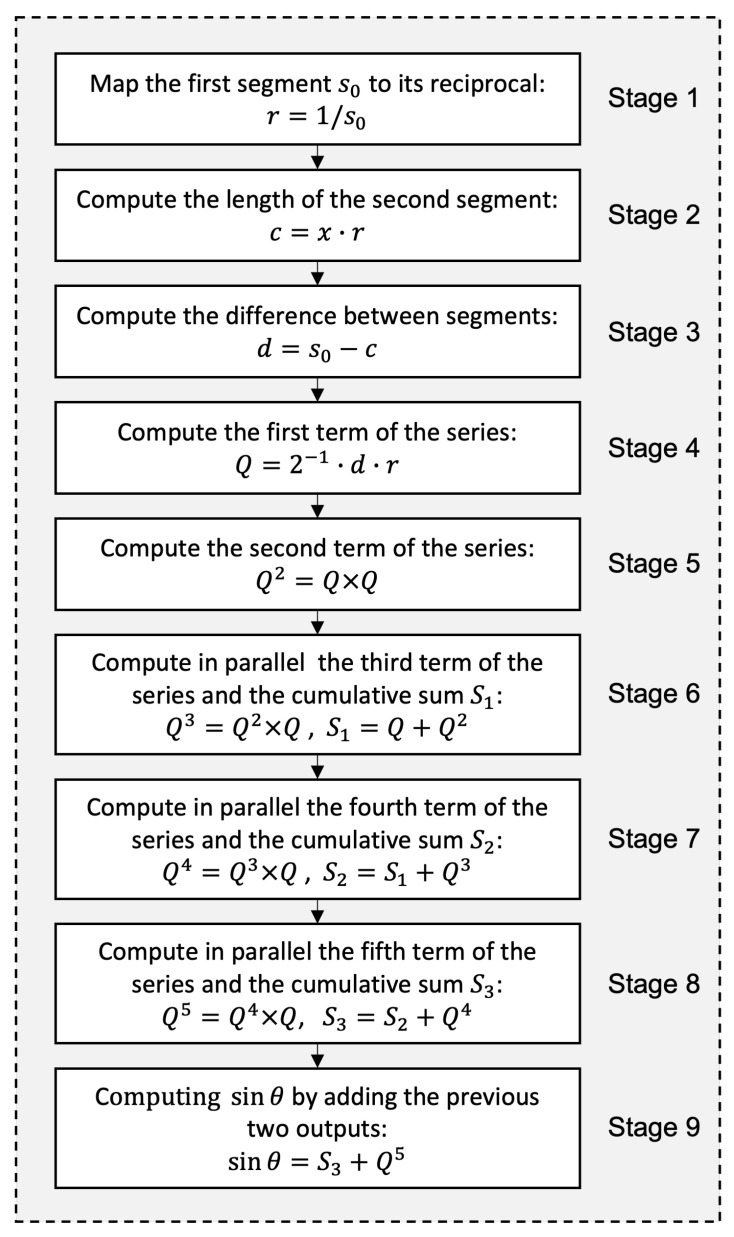
A nine-stage pipeline employed to bypass the two division operations in the quadrature-based method.

The key functionality of the near-lossless compressor is implemented through the division-free quadrature-based method. This method is modeled as a pipeline with nine distinct stages, as depicted in Figure 8. Each stage of the pipeline performs a specific part of the overall computation. The goal is to increase the throughput of the compression system by allowing multiple operations to overlap in execution.

#### 5.2.1. First Stage of the Pipeline

In this stage, a structured array of multiplexers enables rapid access to precomputed reciprocal values, *r*, of $s_{0}$, which are stored in a Q0.16 fixed-point format to balance precision and computational efficiency. This configuration captures the smallest reciprocal value of 8-bit integers, approximately 0.00392, with a 16-bit fractional precision. This precision corresponds to a minimal increment of about 0.0000152587890625, ensuring that small values are accurately represented, thereby minimizing rounding and truncation errors.

#### 5.2.2. Second Stage of the Pipeline

The primary function of the second stage is to compute the product of *x*, which is the area of the rectangle, with the reciprocal of $s_{0}$ to determine the length of the second segment, denoted as *c* in the pipeline. Since the reciprocal is represented in Q0.16 format, multiplying a Q0.16 number by a Q16.0 number (*x*) results in a product with a combined range and precision that may span up to 32 bits.

#### 5.2.3. Third Stage of the Pipeline

The third stage is designed to calculate the difference between the computed segment, *c*, and the seed $s_{0}$, the latter being extended to a similar bit width.

#### 5.2.4. Fourth Stage of the Pipeline

This stage implements Equation (2), where $Q=2^{-1}\times d\times r$ is computed by multiplying the 32-bit difference *d* with the 16-bit reciprocal $r=1/s_{0}$. Such an operation typically requires 48 bits in Q16.32 format. However, the 16 integer bits are unnecessary because $s_{0}$, which represents one side of a rectangle, is usually very close to *c*, which is given by the other side. This proximity ensures that *d*, their absolute difference, is small enough to keep *Q* within the range (−1,1) for nearly all values of *x* in the range $\left[0,2^{16}-1\right]$. The exception occurs when x=3, where $s_{0}=1$, resulting in d=1−(3/1)=−2 and a boundary value of Q=−2/(2×1)=−1. Since Q=−1 does not lead to the convergence of the geometric series, this specific case is handled separately by mapping the final sum directly to the desired value sinθ=−0.5.

#### 5.2.5. Fifth to Ninth Stages of the Pipeline

In the next five consecutive stages (stage 5 to stage 9 of the pipeline), sinθ is computed iteratively by multiplying *Q* by itself and accumulating each successive power of *Q* into a running sum. Once all iterations are complete, the result is truncated to 8 fractional bits, which adequately captures sinθ within its range of [−0.5,0.06]. This level of precision exceeds the 7 bits required to resolve the smallest increment of 0.01 for sinθ. Therefore, retaining 32 fractional bits for all intermediate calculations is sufficient to preserve the desired precision throughout the process.

### 5.3. Postprocessing

Postprocessing aims to produce a more compact representation of $s_{0}$ and its corresponding sinθ values using hardware-friendly, lookup table-based methods. The collective memory requirements of these methods are approximately 1 KB, balancing minimal resource usage with fast and accurate data retrieval. The following subsections detail two key components of the postprocessing step: Golomb–Rice coding and seed indexing logic.

#### 5.3.1. Golomb–Rice Coding

The encoding step leverages a precomputed table to assign compact codes to sinθ values by utilizing the skewed distribution of seed values across sinθ, as illustrated in Figure 3. This distribution guides the code assignment process by first sorting the 29 values of sinθ in descending order of the frequency of their corresponding $s_{0}$ values. Each sinθ value is then assigned a unique index based on its position in the sorted list. At runtime, these indices are used to retrieve the precomputed codes stored in a ROM, enabling efficient and compact encoding.

#### 5.3.2. Seed Indexing Logic

**Figure 9:**
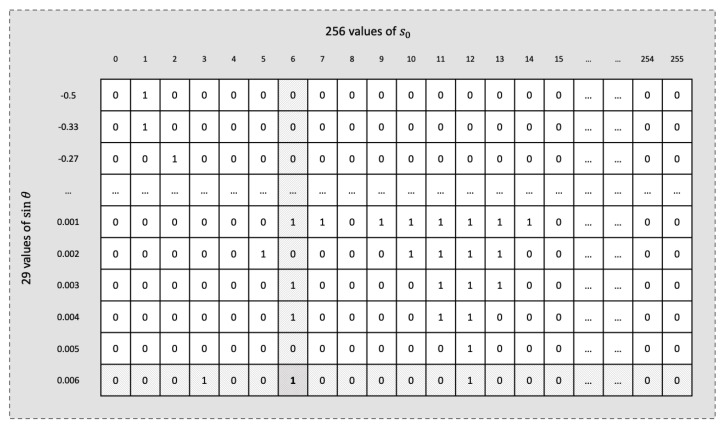
Example illustrating the computation of the index value for the seed ($s_{0}=6$) within the set corresponding to a sinθ of 0.006. A value of 2 for this index is obtained by summing the ones up to bit position 6.

For the seed values, $s_{0}$, a unique identifier (or index) is employed instead of storing the full representation of these values. The index of a value determines its order within the set corresponding to a given sinθ. This could be achieved by constructing a 29×256-bit matrix, implemented as a ROM, where each row represents a specific sinθ value, and each column corresponds to potential values ranging from 0 to 255, as depicted in Figure 9. For each valid value in a set, a binary one is assigned to its respective position in the corresponding row, while unused positions are marked as zero. During retrieval, the row associated with the input sinθ is fetched from the memory, and a bitwise mask of consecutive ones is applied up to the target position. Next, the masked row is divided into 8-bit blocks, and each block is processed using a precomputed LUT to determine the number of ones within that block. The results from the LUT are then summed using a tree-based summation, which efficiently combines the partial results with a logarithmic complexity relative to the number of blocks, that is $O(\log_{2}32)$, in a 256-bit row. The final sum directly corresponds to the order of the input value within the set. This hybrid approach, combining lookup tables and tree summation, minimizes latency and resource usage by leveraging parallelism and hardware-optimized operations.

The primary challenge with this approach lies in its scalability, which is constrained by the substantial amount of required memory. This extensive reliance on memory can impede the ability of the design to efficiently scale to more complex applications. To address this issue, the large lookup tables are replaced by seed indexing logic that leverages observed patterns and gaps within each range of seed values. For instance, consider the scenario when sinθ=−0.06; the corresponding set of seed values is {4, 5, 6, 7, 8, 9, 13, 14, 15, 16}. To map these numbers to their corresponding indices, the logic is structured to subtract 4 if the seed is less than 10; otherwise, 7 is subtracted to compute the index, leading to a new set of successive numbers in the range 0-9. Furthermore, the most complex set encountered required no more than five logic levels involving deeply nested conditional statements. All indexing logic blocks are designed to operate within the clock period of the design.

## 6. Results and Discussions

In this section, we present the results obtained using a comprehensive suite of tools and hardware configurations. Programming and simulation tasks were performed with Quartus Prime and ModelSim, respectively, utilizing VHDL as the programming language. Our design targets a Cyclone V FPGA, model 5CGTFD9E5F35C7, chosen for its higher number of I/O pins compared to other models in the Cyclone V series. This feature is crucial as it allows us to replicate the design 19 times, enabling a compression engine with 19 units working in parallel, significantly enhancing the compression performance. The analyses were supported by a computer equipped with an Intel(R) Core(TM) i7-10510U CPU, clocked at 1.80 GHz (up to 2.30 GHz), and 16 GB of RAM while running a Windows 11 Operating System. We define the measure of efficiency to be equal to the ratio of throughoput divided by the power requirement (in MSps/Watt). The results provided below illustrate the efficiency and performance capabilities of the proposed method and the hardware configurations employed.

### 6.1. Resource Utilization and Scalability

As indicated by the Quartus Prime compilation report, the proposed FPGA design utilizes 10,320 out of 113,560 available Adaptive Logic Modules (ALMs), which account for 9% of the total logic resources. Additionally, 114 out of 342 available Digital Signal Processors (DSPs) are employed, constituting about 33% of the total DSP capacity. The design also makes use of 6873 registers, which, in combination with Block Randon Access Memory (BRAM) utilization of 73,872 out of a total of 12,492,800 (around 1%), highlight the efficient use of memory resources. On the other hand, 553 out of 616 available pins are used, equating to 90% utilization. The low usage of logic resources, alongside moderate DSP and minimal memory utilization, suggests that this design could achieve greater scalability by addressing the current bottleneck through strategic hardware upgrades.

### 6.2. Clock Frequency

The maximum operating frequency of our system, as reported by the compilation report, is 84.83 MHz. This frequency maintains a positive timing slack, indicating that the system is configured for optimal speed without compromising stability.

### 6.3. Throughput

Out of 560 available General-Purpose IO pins (GPIOs), 553 are actively utilized on the Cyclone V FPGA to support 19 independent processing units. Each of these units manages to process one full 16-bit sample every clock cycle. As a result, the aggregate throughput for the system reaches 19 samples each cycle. With the system operating at a maximum frequency of 84.83 MHz, this translates to an overall system throughput of 1611.77 MSps.

### 6.4. Power Requirement

By configuring the optimization technique of the compiler for optimal performance, the power requirement was measured at 0.886 Watts. This achieved value demonstrates the dual benefit of our approach, enhancing system performance while ensuring energy efficiency. Both are crucial for sustainable operation in real-world applications.

### 6.5. Comparison with Division-Based Approach

We compare the proposed division-free approach with its division-based counterpart, that is, the direct implementation of Algorithm 1 without optimization. Both implementations were evaluated with similar hardware configurations to highlight the performance benefits of eliminating the two division operations. Since the number of instances is limited by the available I/O pins, this constraint limits both the division-based and division-free approaches to 19 instances of the pipeline running in parallel. Table 3 summarizes the key performance metrics, including clock rate, throughput, power requirement, and efficiency for both approaches.

We observe that the division-free approach achieves significantly higher throughput, reaching 1611.77 MSps compared to 394.6 MSps for the division-based method, thus providing an enhancement of 308.46%. This improvement is primarily due to the elimination of direct division operations, which reduced the clock period from 51 ns to 19 ns in the division-free approach. This translates to a clock rate increase of 308.43%.

In terms of power requirement, the division-based method relies heavily on logic resources, while the division-free approach reduces logic usage by incorporating DSPs. This trade-off between logic and DSP utilization nearly balances the overall power requirement despite their differing resource usage strategies. It results in a slight increase in power requirements by 13.15% for the division-free approach.

Efficiency, measured as throughput per Watt, further underscores the advantages of the division-free design. With an efficiency of 1819.15 MSps/Watt, the method outperforms the division-based implementation, which achieves 504 MSps/Watt, by 260.94%. This represents an improvement of nearly four times, emphasizing the significant optimization achieved by the division-free approach in FPGA-based compression of hyperspectral images.

### 6.6. Comparison with State-of-the-Art Implementations

We next present a comparative analysis with the latest FPGA-based hyperspectral image compression implementations. Table 4 below outlines key performance metrics that include throughput, power requirement, and efficiency in various studies. Efficiency metrics, calculated as throughput per Watt of power, provide insight into how effectively each system uses power to process data.

The throughput values across the selected studies span a wide range, from 12.5 to 1375 MSps, illustrating the different architectural choices and optimizations. On the higher end, the Xilinx Kintex Ultrascale XCKU040 achieves a throughput of 1375 MSps while consuming 4.221 Watts, illustrating a potential trade-off between performance and power efficiency. The Cyclone V FPGA used in this work achieves the lowest power requirement of just 0.886 Watts while delivering a significant throughput of 1611.77 MSps. The highest efficiency value of 1819.15 MSps/Watt is also achieved by the division-free approach employed in this study, suggesting a highly efficient use of power.

The resource utilizations for each implementation are detailed in Table 5 showing the allocation of LUTs, Flip-Flops (FFs), DSPs, and BRAMs. The analysis of this table is essential for understanding how resource allocation impacts overall system performance and efficiency. We observe from the table that high DSP utilization correlates strongly with enhanced performance. Both [37] and this work demonstrate that significant DSP usage contributes to higher throughput rates. Additionally, the elevated power requirement noted in the aforementioned study can be attributed to the significant use of both LUTs (nearly 6.5 times) and FFs (10 times) when compared to our design. In our design, we significantly reduced reliance on BRAMs, utilizing only 23 units. This strategic reduction is achieved by replacing a large, BRAM-intensive lookup table, shown in Figure 9, with a custom-designed logic for indexing seed values. This adjustment substantially lowers BRAM demand and enhances the scalability of our system, allowing it to maintain high throughput while accommodating future enhancements and applications.

## 7. Conclusions

In this paper, we have implemented an innovative FPGA-based near-lossless compression technique for hyperspectral images, leveraging the previously developed quadrature-based square rooting method. This method is specifically optimized for addressing challenges associated with limited power and computational resources, which are particularly critical onboard satellites. The division-free approach utilized here is well-suited for real-time processing applications due to its efficient design. The proposed technique achieved a high throughput of 1611.77 MSps while maintaining a low power requirement of less than a Watt when compared with state-of-the-art implementations. The modest utilization of logic resources and the concomitant moderate number of DSP blocks employed in this design suggest the potential for enhanced scalability. However, the current bottleneck, illustrated by the limited number of available pins, needs to be addressed through strategic hardware upgrades.

Future work could further refine the quadrature-based square rooting method to enhance compression performance. Firstly, improving compression ratios would make it more efficient for environments with strict storage and bandwidth limitations. Further, implementing this compression technique across different FPGA platforms and its validation could provide valuable insights into performance and energy consumption variations. Additionally, integrating security features such as encryption into the compression process would protect sensitive data, which are especially important when data security is critical. These suggestions aim to broaden the scope of this research and enhance its practical applications.

## Figures and Tables

**Table 1:** Comparison of lossless and near-lossless compression results, which highlight the improvement in reduction percentage with MRE and PSNR values for the near-lossless approach. The scenes are taken from the publicly available Corpus dataset.

Imager	Scene	Lossless [40]	Near-Lossless	MRE	PSNR (dB)
AVIRIS	YS *_sc00_uncal	22%	38.60%	0.0102	50.95
AVIRIS	YS_sc03_uncal	24%	38.60%	0.0101	51.08
AIRS	gran9	25%	38.50%	0.0100	50.61
AIRS	gran16	26%	38.60%	0.0100	50.57
AIRS	gran60	20%	38.50%	0.0100	50.74
AIRS	gran82	32%	38.60%	0.0100	50.48
AIRS	gran120	27%	38.50%	0.0100	50.61
AIRS	gran126	22%	38.50%	0.0100	50.68
AIRS	gran129	34%	38.60%	0.0100	50.65
AIRS	gran151	26%	38.50%	0.0101	50.66
AIRS	gran182	22%	38.50%	0.0101	50.71
CASI	t0180f07	18%	38.50%	0.0100	50.23
CASI	t0477f06	27%	38.80%	0.0100	50.28
CRISM	sc182	37%	38.60%	0.0100	50.69

* YS: yellowstone.

**Table 2:** Maximum values of *x* used as pivots in the binary search algorithm for each binary representation length *n*, along with the corresponding shift amounts, which are calculated as ⌊n/2⌋.

Number of Bits (*n*)	Pivot	Shift Amount (⌊n/2⌋)
16	65535	8
15	32767	7
14	16383	7
13	8191	6
12	4095	6
11	2047	5
10	1023	5
9	511	4
8	255	4
7	127	3
6	63	3
5	31	2
4	15	2
3	7	1
2	3	1
1	1	0

**Table 3:** Performance metrics comparison between the division-based and division-free implementations. A positive value in relative enhancement means that the division-free approach yielded more improvement.

Metric	Division-Based Approach	Division-Free Approach	Relative Enhancement (%)
Clock rate (MHz)	20.77	84.83 *	308.43
Throughput (MSps)	394.6	**1611.77**	308.46
Power (Watts)	**0.783**	0.886	−13.15 **
Efficiency (MSps/Watts)	504	**1819.15**	260.94

* Bold faced values indicate the best results. ** The negative value represents an increase in power requirement of 13.15% for the division-free approach.

**Table 4:** Performance metrics of FPGA-based implementations of HSI near-lossless compression and their comparison with our work using the division-free approach.

FPGA Platform	Compression Method	Throughput (MSps)	Power (Watt)	Efficiency (MSps/Watt)	Instances	Reference
Xilinx Kintex UltraScale	CCSDS 123.0-B-2	12.5	2.48	5	NA	[35]
NA	CCSDS 123.0-B-2	125	-	-	NA	[36]
Xilinx Kintex Ultrascale XCKU040	CCSDS 123.0-B-2	1375	4.221	325.8	5	[37]
Xilinx Kintex Ultrascale XCKU040 SRAM	CCSDS 123.0-B-2	305	1.525	200	NA	[38]
Virtex-7 VC709 with XCKU040	CCSDS 123.0-B-2	249.6	1.2	208	NA	[39]
Cyclone V FPGA 5CGTFD9E5F35C7	Quadrature Based	1611.77 *	**0.886**	**1819.15**	19	This work

* Bold faced values indicate the best results.

**Table 5:** Resource utilization of different FPGA-based implementations of HSI near-lossless compression and their comparison with this work.

Reference	LUTs	FFs	DSPs	BRAMs
[35]	17,185	11,915	63	85
[36]	-	-	-	-
[37]	67,362	68,987	115	306
[38]	5058	3322	17	1
[39]	17,390	16,135	30	443.5
This Work	10,320	6873	114	23

## Data Availability

The original contributions presented in the study are included in the article, further inquiries can be directed to the corresponding author.
